# Effect of idol immersion on water quality and Tilapia fish in Futala, Gandhisagar and Ambazari lakes of Nagpur, India

**DOI:** 10.1186/2193-1801-3-669

**Published:** 2014-11-12

**Authors:** Manisha D Giripunje, Abhay B Fulke, Pravin U Meshram

**Affiliations:** Department of Environmental Science, Sevadal Mahila Mahavidyalaya, Nagpur, 440009 India; Council for Scientific and Industrial Research-National Environmental Engineering Research Institute (CSIR-NEERI), Nagpur, 440020 India

**Keywords:** Idol immersion, Heavy metals, Water quality, Tilapia fish (*Oreochromis mossambicus*)

## Abstract

**Introduction:**

Idol immersion activity is one of the sources of heavy metal pollution in the lakes of India. Futala, Gandhisagar and Ambazari lakes of Nagpur city are highly involved with idol immersion activity.

**Case description:**

In this study, water and Tilapia fish (*Oreochromis mossambicus*) of Futala, Gandhisagar and Ambazari lakes were analyzed for heavy metals by using inductively coupled plasma optical emission spectrometry (ICP-OES).

**Discussion and evaluation:**

The results were high compared with the Bureau of Indian Standards (BIS) for water quality and Food and Agriculture Organization (FAO)/World Health Organization (WHO) for fish. The results showed appreciable high levels of heavy metal as lead, cadmium, copper, iron and manganese in water and fish in study lakes. It was observed that Gandhi Sagar lake was more contaminated after the idol immersion activity. Concentrations of Pb and Cd in fish of Gandhisagar lake were found high levels, ranged 0.83 and 0.47 μg/g respectively.

**Conclusion:**

The results of the present investigation indicate the unsafe condition for human consumption and environmental health.

## 1. Introduction

Water is a vital part of our life. However, nowadays contamination of water is a serious global issue. According to European Environmental Agency the average person has consumed some 150–200 litres of fresh water and the USA almost uses 500 billion litres of fresh water per day to cool electric power plants, and same amount to irrigate crop fields. In India also irrigation & industrial use of water much more than water used for drinking purpose. Water pollution is turning significant with respect to human health and food security. Water pollution occurs due to industrial waste water and urban sewage into water bodies. Additionally, religious activities near the banks of water bodies become a threat to the ecosystem. India is a country of rituals and idol immersion activity is major anthropogenic activity causing water pollution in different water bodies such as lakes, reservoirs, ponds, rivers, canals and seas (Bajpai et al. 2002). These idols generally made up of clay, textiles, bamboo and non-degradable materials including plastic, cement, plaster of Paris (PoP), paints, varnishes and toxic dyes, and also decorated with various polishes, ornaments and cosmetic items (Upadhyaya and Bajpai 2010; Bajpai et al. 2002; Shukla 2004). The chemical paints used on these idols contain heavy metals as lead, cadmium, copper, iron, manganese, mercury, zinc, chromium, arsenic and various organic and inorganic materials, leading to alteration in water quality. After decomposition, the biodegradable matter recycles and non-biodegradable substances form sediments. The bio-accumulation of heavy metal transfers toxic element from producer to consumer level and health hazard for consumers (Kaur et al. 2013; Mukerjee 2005; Storelli et al. 2005; Reddy et al. 2012). Idol immersion caused contamination of noxious dyes in lakes and ultimately affects food crops, as this water used for irrigation. Hence, after idol immersion, heavy metals pass into the food chain from fish to human beings increases that have a particular significance in eco-toxicology (Reddy and Kumar 2001). Fish is significant indicators in freshwater systems for the estimation of heavy metal pollution level because it is an important food source for human and organisms of high trophic level in the food chain (Abdel-Baki et al. 2011; Agah et al. 2009; Blasco et al. 1998 and Rashed 2001). Significant positive correlations were observed levels of heavy metal in lakes and heavy metal accumulation in aquatic organisms (Farkas et al. 2003). Similarly, the toxicity of a heavy metal is not determined by the concentration, but forms also have influence on toxicity (Baird and Cann 1995). Immersion of idols is a source of pollution; deteriorate lakes and rivers of India. The current study was conducted to show freshwater lakes as models to find out the contamination of chemical pollutants contributed through idol immersion activity. Immersed non-degradable materials contaminate the lake water and bio-accumulate the heavy metals in the biological system, transfer the toxic elements from primary producers to consumers to have an influence on human health. These are highly sensitive problem and attempting to deal with it (Ujjania and Multani 2011).

**Figure 1:**
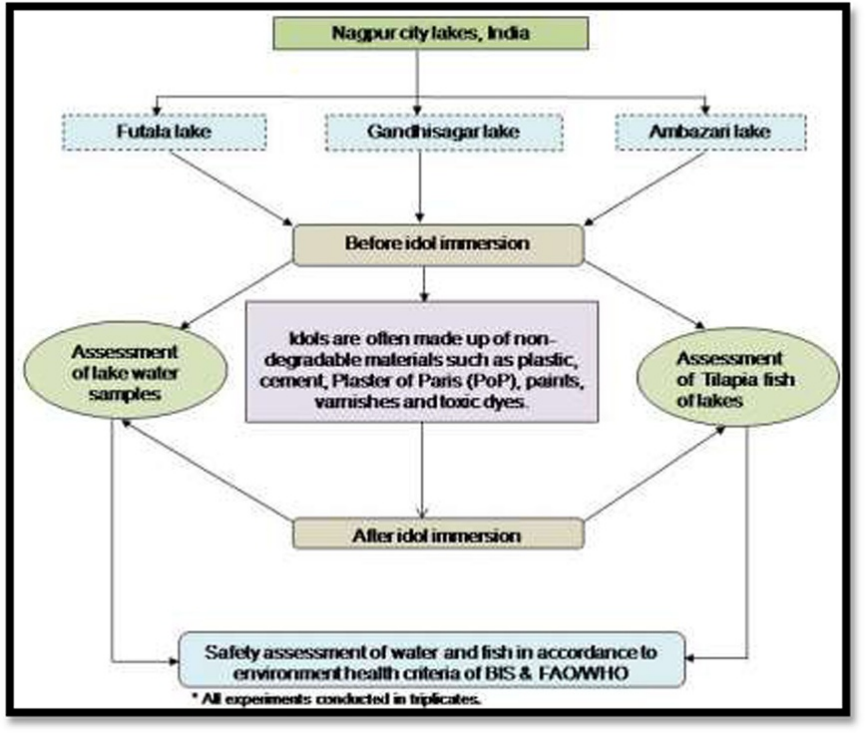
**Flow-sheet for the assessment study of water quality and Tilapia fish after idol immersion activity.** Note: BII-Before idol immersion, AII-After idol immersion.

In this study, water and Tilapia fish (*Oreochromis mossambicus*) were analyzed for heavy metals by using inductively coupled plasma optical emission spectrometry (ICP-OES). The results were compared with Bureau of Indian Standards (BIS) for water quality and Food and Agriculture Organization (FAO)/World Health Organization (WHO) for fish. A flow-sheet depicting the work flow is given in Figure 1. The need of study occurred from unsafe idol immersion activity in India, causes unknowing threat to human consumption from water and fish of the lakes. Such pollution activities exist in all over India for many years. However, this is a single study comprehensively analyzed on idol immersion activity. The aim of the study is to focus on a risk assessment of the potential idol immersion activity for undesirable health effects because of the ingestion of heavy metal from fish in the lakes. Fish is an important source of metal exposure that undertaking a risk assessment seems to be justified.

## 2. Material and methods

### 2.1. Study sites

**Figure 2:**
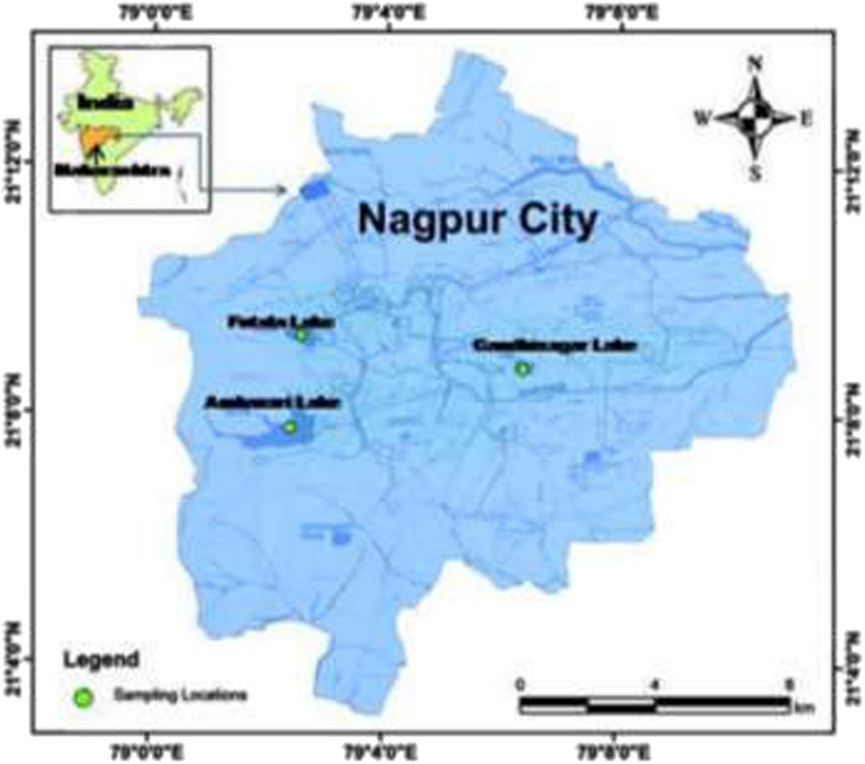
**Locations of Lakes of the Nagpur, Inset Maharashtra, India.**

The study was conducted in three lakes of Nagpur city: Futala, Gandhisagar and Ambazari (Figure 2). Futala lake is situated at latitude of 21′09′11.74″ N and longitude 79′02′32.77″ E. The catchment area of the lake is 0.40 sq. Kms. Futala lake built by King Bhosle dates back centuries. Gandhisagar lake is situated at latitude of 21′8′44.82″ N and longitude 79′5′59.50″ E. The catchment area of Gandhisagar lake is 0.181 Sq. Kms. Gandhisagar lake was established as a source of water supply by Chand Sultan, the ruler of Nagpur, India in the year 1737. Ambazari lake lies at lat. 20′35′21.44″ N and long. 78′15′79.40″ E. It is the largest lake in the city and catchment area is 1.185 Sq. Kms. Ambazari tank supplies the drinking water to the Nagpur city. Futala, Gandhisagar and Ambazari lakes practices for irrigation and commercialized fishing.

### 2.2. Sample collection

Water samples and Tilapia (*Oreochromis mossambicus*) fish were collected from Futala, Gandhisagar and Ambazari lakes in Nagpur, India. The samples were collected at different intervals i.e. before and after two months of the idol immersion activities. Water samples were taken in sterilized sampling bottles, below 10 to 20 cm of the surface from study sites. Fishes were taken using a drag net and mean total lengths, total weights of fish were measured as (17.80 cm and 65.38 g) in Futala lake, (17.35 cm and 72.63 g) in Ambazari lake and (17.10 cm and 79.65 g) in Gandhisagar lake. Fish samples were placed on ice immediately and taken to the laboratory, where the samples were deep frozen at −20°C until they could be prepared for digestion and analysis. The experimental research on fishes was ethically approved by Animal Ethics Committee of Maharashtra Animal and Fisheries Sciences University, Nagpur.

### 2.3. Chemical analysis of water samples

Water samples were subjected to chemical analysis with the prescribed procedures of dissolved oxygen (DO), Total hardness, sulphate, calcium and magnesium. The temperature of the water samples measured using centigrade thermometer on the lake sites. DO was estimated on the site by Wrinkler method and Total hardness, Ca and Mg estimated by using EDTA complex metric titration and Sulphate determined by barium chloride and measured with Spectrophotometer (APHA 2005).

### 2.4. Heavy metal analysis of water and fish samples by ICP-OES

Heavy metals as lead, cadmium, copper, iron and manganese were selected for the study. Samples digested and analyzed by using inductively coupled plasma optical emission spectrometry (ICP-OES) (Perkin Elmer, Optima 4100DV) according to the method described in APHA (2005) and USDA (2008). Before the digestion of samples, glasswares used for the experiment, rinsed by 10% (v/v) nitric acid and deionized water. Fish samples were thawed and muscles of fish removed with sterilized surgical blades and scissor. Fish muscles oven dried at 80°C in acid washed petri dishes upto a constant weight. Fish samples were kept in dessicators for cooling. Fish muscle homogenized with mortar and pestel and ground to fine powder and weighed. Moisture content of individual fish sample calculated. 0.5 g of fine powdered muscle sample processed in duplicate and then digested using closed-vessel microwave digestion (Milestone model Start D, Italy). Fish muscles were digested in Nitric acid and subjected to follow four steps of microwave digestion program (Table 1). After digestion, 2 ml of 30% hydrogen peroxide added to digests because it reduces vapours of nitric acid and accelerate the organic substance digestion by increasing the temperature (Dig-Acids 2001). Blanks are used for the authentic determination of analysis. For analytical quality, fish samples analysed in triplicates. The digested fish samples were diluted with 50 ml ion-free water in acid washed standard flasks and each fish sample filtered through 0.45 μm Whatman filter paper. After filtration, digested samples were analyzed using ICP-OES. Operational parameter settings of ICP-OES (Perkin Elmer, Optima, 4100DV) were shown in Table 2. Multi-elemental standard solutions (Merck) used for the standardization, and prepared by diluting stock solutions of 1000 mg/L (Mohammed 2007). ICP-OES detection limits for lead is 220.373 nm, cadmium- 214.438 nm, copper- 324.653 nm, iron- 238.204 nm and manganese-238.204 nm. The certified standard reference material for fish (SRM 1577b) from National Institute of Standards and Technology, USA (NIST) was used. The levels of heavy metals in fish were expressed as μg/g wet weight. All experiments were done in triplicates and the mean along with standard deviation of the experimental results was calculated using Microsoft Excel.

**Table 1:** **Microwave digestion program used for Fish (Source: USDA 2008)**

Steps	Temperature	Time	Power
1	25-96 C	20 min	1000 W
2	96 C (Hold)	30 min	1000 W
3	180 C	10 min	1000 W
4	180 C (Hold)	10 min	1000 W

**Table 2:** **Summary of the operational parameter settings used for the ICP-OES (Perkin Elmer, Optima 4100DV)**

Characteristics	Instrument condition
RF Generator	Fully Solid-state generator. Operating frequency-40 MHz
RF Power	Adjustable power from 750 to 1300 watts
Spray chamber	Scott type
Nebulizer	Cross Flow
Plasma gas flow	15.0 L/min
Auxiliary gas flow	L/min
Nebulizer gas flow	0.60 L/min

## Result and discussion

### 3.1 Chemical parameters in water

Results for water samples of three lakes before and after idol immersion were illustrated in Table 3. DO concentration is a common indicator of the health of the aquatic ecosystem. In the present study, before and after immersion of idols, DO values were observed from 4.09 to 5.29 and 0.89 to 2.34 mg/L respectively in three lakes of Nagpur city during a span of two months. The results showed low levels of DO i.e. 0.89 to 2.34 mg/L after idol immersion in lakes of Nagpur. Similar observations were also made by Varughese et al. (2004) in the urban eutrophic lake of Bhopal, India. In this study, calcium and magnesium salt levels were increased after idol immersion in three study lakes of Nagpur. However, levels of calcium and magnesium were found below the limits of BIS. Reddy and Kumar (2001) informed that magnesium is non-poisonous but increases the hardness of water whereas Wetzel in 1983 reported that increase in salt content declines oxygen solubility exponentially in the water bodies. In the current study, total hardness values were observed from 78.12 to 154.35 mg/L and 198.10 to 275.70 mg/L before and after idol immersion respectively in three study lake sites that indicated the elevation in hardness of water after idol immersion in three lakes of Nagpur city. However, the total hardness remained well below the BIS recommended limit of total hardness for drinking water that is reported to be 300 mg/L. Vyas et al. (2006) reported that the total hardness in the upper and lower lakes of Bhopal to be in the range of 32-96 mg/L before and 96-198 mg/L after idol immersion. Rose and Cravotta (1998) reported that, sulphate is a primary constituent of the effluent waste. However, idols are made of PoP, the major source of sulphate. In the current study, sulphate values were found from 43.9 to 69.2 and 144.5 to 187.3 mg/L before and after idol immersion respectively in three study sites, indicating increase in the sulphate after idol immersion. However, levels of sulphate were found below the limit of BIS. Low range of sulphate in water is non-toxic but, high amount of sulphate may result in dehydration, diarrhea and intestinal discomfort (Rose and Cravotta 1998). Sulphate concentrations (160.1,187.3 and 144.5 mg/L) after idol immersion in water samples were about 3.2, 2.7 and 3.2 folds high as compared to before idol immersion in Futala, Gandhisagar and Ambazari lakes respectively.

**Table 3:** **Chemical parameters of the water samples in various lakes at Nagpur**

Lakes		DO	TH	Sulphate	Ca	Mg	Pb	Cd	Cu	Fe	Mn
Futala	BII	4.25	78.12	50.2	44.2	7.08	0.076	0.024	0.483	0.125	0.060
	AII	1.21	198.10	160.1	68.4	10.02	0.093	0.053	0.717	0.220	0.087
Gandhi Sagar	BII	4.09	103.60	69.2	54.3	9.63	0.081	0.034	0.522	0.185	0.076
	AII	0.89	221.30	187.3	78.1	15.16	0.105	0.079	0.839	0.342	0.094
Ambazari	BII	5.29	154.35	43.9	40.02	19.23	0.046	0.019	0.329	0.103	0.050
	AII	2.34	275.70	144.5	61.9	28.01	0.074	0.048	0.625	0.189	0.074
PL	-	4*	300	400	75	30	0.05	0.01	1.5	1.0	0.1

Note: BII-Before idol immersion, AII-After idol immersion. All values in mg/L. PL-Permissible limit of BIS in mg/L.DO-Dissolved Oxygen, TH- Total Hardness, *Tolerance limits (ISI-IS: 2296) for inland surface waters in India, class – d, e.

### 3.2. Metals in water

**Figure 3:**
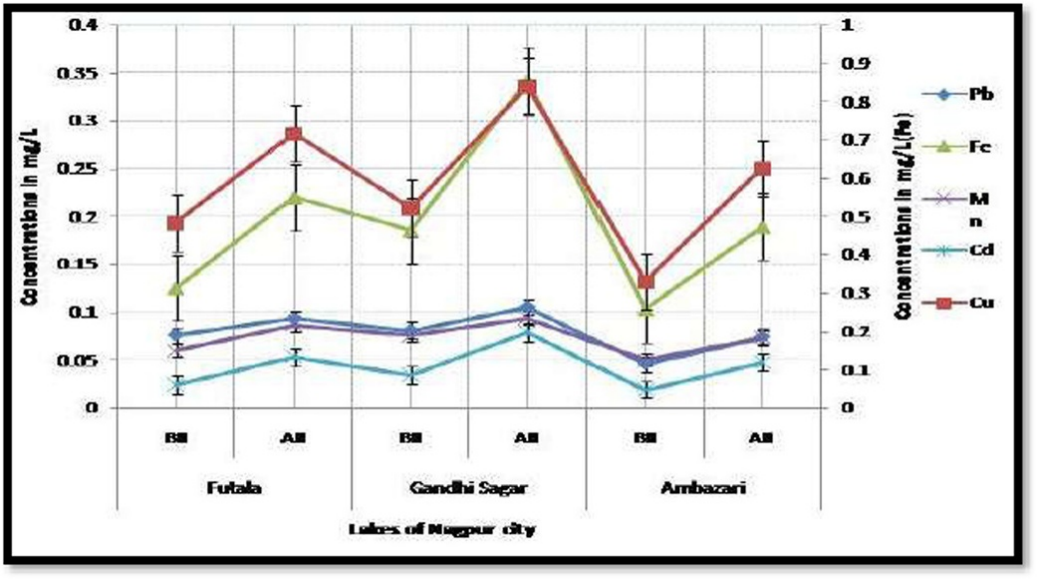
**Heavy metals in water samples before and after idol immersion in various lakes**

The concentrations of heavy metals in water samples of three lakes were observed from 0.046 to 0.081 and 0.074 to 0.105 mg/L for lead, 0.019 to 0.034 mg/L and 0.048 to 0.079 mg/L for cadmium, 0.329 to 0.522 mg/L and 0.625 to 0.839 mg/L for copper, 0.103 to 0.185 mg/L and 0.189 to 0.342 mg/L for iron, 0.050 to 0.076 mg/L and 0.074 to 0.094 mg/L for manganese before and after idol immersion respectively. Concentrations of metals such as lead, cadmium, copper, iron and manganese had increased noticeably after idol immersion into three lake water samples (Figure 3) compared with specifications of highest desirable limits set by BIS standards (Table 3). This is due the chemical paints used on these idols contain heavy metals as lead, cadmium, copper, iron, manganese, mercury, zinc, chromium, arsenic and various organic and inorganic matter, leading to alteration in water quality.Upadhyaya and Bajpai (2010) reported that, the effect of idol immersion on the lakes of Bhopal showed a significantly higher side for lead metal concentration after idol immersion activity Bajpai et al. (2002) pointed that the possible source of increasing lead metal in lakes resulting from the immersion of idols during the festival season, domestic sewage and effluent discharge from waste disposal sites. Levels of cadmium in water samples were found alarmingly above permissible limits of BIS after idol immersion in three study lakes of Nagpur. Cadmium is a non-essential metal but toxic even when present in very low concentration. Toxicity of cadmium exacerbated by the fact, has an extremely long biological half life. Therefore, cadmium retained for long periods of time in organisms after bioaccumulation (Larison et al. 2000). In the current study, copper found in higher concentrations after idol immersion in lakes of Nagpur. Levels of copper in water samples were found below permissible levels of BIS. Puttaiah and Kiran (2007) reported that, copper is essential metal and not expect to cause any damage to aquatic ecosystem. However, excess copper that is above 1.5 mg/L cause hemolytic anemia and kidney damage (WHO 1989). This has significance in human eco-toxicological studies.

### 3.3 metals in fish muscles

**Figure 4:**
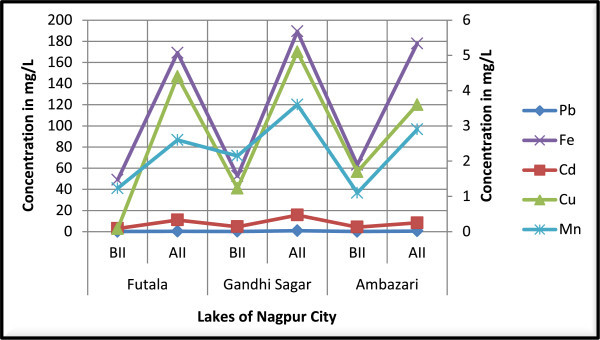
**Heavy metals in tilapia (** ***Oreochromis mossambicus*** **) fishes tissues before and after idol immersion in various lakes.**

Tilapia fish (*Oreochromis mossambicus*) samples of Futala, Gandhisagar and Ambazari lakes, Nagpur were analyzed for the concentrations of heavy metals such as lead, cadmium, copper, iron and manganese, increased noticeably after immersion of idols (Table 4 and Figure 4). Values were compared with specifications of limits set by FAO/WHO standards. In this study, levels of lead and cadmium in fish muscles were recorded in Futala lake (0.14 ± 0.24, 0.32 ± 0.89 and 0.09 ± 0.33, 0.33 ± 0.58 μg/g wet wt.), Gandhisagar lake (0.19 ± 0.29, 0.83 ± 0.76 and 0.14 ± 0.51, 0.47 ± 0.80 μg/g wet wt.) and Ambazari lake (0.15 ± 0.20, 0.56 ± 0.34 and 0.13 ± 0.62, 0.25 ± 0.28 μg/g wet wt.) before and after idol immersion respectively. Concentrations of lead and cadmium in fish muscle tissues of all three sites were compare with permissible levels (FAO/WHO 1999). The levels were found above the permissible limits after idol immersion. Dreisbach and Robertson (1987) and Lee (1977) reported levels of lead and cadmium in muscle tissues of Tilapia nilotica fish to be at baseline for human consumption (Lead, 2 ppm and Cadmium, 1.5 ppm). However, Eisler in 1988 and Weber and Dingel in 1997 reported that lead is a neurotoxin, causes behavioral deficits in vertebrates and adversely affects survival, growth, learning rates, and metabolism. Similarly, Mohammad et al. 2013 reported lead toxicity causing spinal deformity and blackening of the caudal region. Copper concentrations were found in fishes of Futala lake (0.08 ± 0.83, 4.40 ± 0.72 μg/g wet wt.), Gandhisagar lake (1.23 ± 0.42, 5.10 ± 0.67 μg/g wet wt.) and Ambazari lake (1.70 ± 0.75, 3.60 ± 0.58 μg/g wet wt.) before and after idol immersion respectively. Levels of Copper in fish samples were found alarmingly above the permissible level (FAO/WHO 1999) after idol immersion in three study lakes of Nagpur. Copper concentrations (4.40 ± 0.72, 5.10 ± 0.67 and 3.60 ± 0.58 μg/g wet wt.) after idol immersion in Tilapia fish were about 55, 4, and 0.5 folds high in comparison to before idol immersion in Futala, Gandhisagar and Ambazari lakes respectively. However, Sivaperumal et al. (2007) stated that, copper is an essential element that serves as a co-factor in a number of enzymes systems and is necessary for the synthesis of hemoglobin, but very high intake of Cu cause adverse health problems for living organism. In this study, Iron levels in fish were found (49.0 ± 0.33 and 169.1 ± 0.82 μg/g wet wt.) for Futala lake, (52.5 ± 0.37 and 189.3 ± 0.61 μg/g wet wt.) for Gandhisagar lake and (63.2 ± 0.50 and 177.9 ± 0.10 μg/g wet wt.) for Ambazari lake before and after idol immersion respectively. As a whole, Iron concentrations were observed above the permissible level according to the FAO/WHO (1999). The highest degree of iron level was found in fish sample of Gandhisagar lake after idol immersion. Iron concentration was found higher than concentrations of lead, copper, cadmium and manganese in fish muscles. However, Kumar et al. (2011) reported that, iron was found to be the most abundant metal in muscle tissue of fish. Rajkowska and Protasowicki in 2012 reported that manganese is absorbed through the gills and muscles of fish. However, in our study, manganese values in fish muscles were found in Futala lake (1.23 ± 0.49, 2.60 ± 0.67 μg/g wet wt.), Gandhisagar lake (2.15 ± 0.23, 3.60 ± 0.77 μg/g wet wt.) and Ambazari lake (1.10 ± 0.59, 2.90 ± 0.94 μg/g wet wt.) before and after idol immersion respectively. Manganese levels were above the permissible level (FAO/WHO 1999) after idol immersion in all study lakes of Nagpur. The highest degree of manganese level was found in fish sample of the Gandhisagar lake after idol immersion. However, Ambedkar and Muniyan (2012) reported that, manganese is an essential micronutrient and does not occur in naturally as a metal in aquatic ecosystems. Kumar et al. (2011) stated that, essential metals such as iron, copper and manganese were found to be at higher levels in fishes, this could be due to their utility as co-factors in the activation of enzymes and normalized to retain a definite homeostatic position in fishes. Kumar et al. also reported that non-essential metals such as lead and cadmium have no biological utility and their concentrations in fishes are normally low. In the present study, Similar results were also found before idol immersion but noticeably high levels of heavy metals in fishes were found after idol immersion.

**Table 4:** **Heavy metal concentrations of fish in various lakes in Nagpur**

Lakes	Metals	Pb	Cd	Cu	Fe	Mn
Futala	BII	0.14 ± 0.24	0.09 ± 0.33	0.08 ± 0.83	49.0 ± 0.33	1.23 ± 0.49
	AII	0.32 ± 0.89	0.33 ± 0.58	4.40 ± 0.72	169.1 ± 0.82	2.60 ± 0.67
Gandhi Sagar	BII	0.19 ± 0.29	0.14 ± 0.51	1.23 ± 0.42	52.5 ± 0.37	2.15 ± 0.23
	AII	0.83 ± 0.76	0.47 ± 0.80	5.10 ± 0.67	189.3 ± 0.61	3.60 ± 0.77
Ambazari	BII	0.15 ± 0.20	0.13 ± 0.62	1.70 ± 0.75	63.2 ± 0.50	1.10 ± 0.59
	AII	0.56 ± 0.34	0.25 ± 0.28	3.60 ± 0.58	177.9 ± 0.10	2.90 ± 0.94
PL**	-	0.214	0.1	3.0	43.0	2.0-9.0

Note: BII-Before idol immersion, AII-After idol immersion. PL: Permissible limits (wet weight ** μg/g) according to (FAO/WHO 1999).

## 4. Conclusion

The study suggests that idol immersion has major impact on water quality and fish of the lakes, particularly concerning heavy metals. Heavy metal concentrations in lake water and muscles of Tilapia fish (*Oreochromis mossambicus*) increased after immersion of Idols compare with the maximum permissible concentrations for human intake. Usually, humans are exposed to these accumulated metals in fish by ingestion and can impose higher risks of human health because fish found virtually everywhere in the aquatic environment and they play a major ecological role in the aquatic food webs because of their function as a carrier of energy from lower to higher trophic levels. Hence safe eating guidelines should be in practice to inform people about the recommended level of consumption for fish caught in such lake water. It is also recommended that the idols should be used that are made up of natural biodegradable materials instead organic pollutant materials in natural water bodies for eco-friendly customs. Henceforth, this reduce the chances of spoiling the aquatic eco-system and also public health and governmental bodies strictly deal with this issue for the sake of the environment and public health by promoting the use of nature friendly biodegradable materials to create idols.
